# Tracking of eating patterns and overweight - a follow-up study of Norwegian schoolchildren from middle childhood to early adolescence

**DOI:** 10.1186/1475-2891-10-106

**Published:** 2011-10-06

**Authors:** Inger M Oellingrath, Martin V Svendsen, Anne Lise Brantsæter

**Affiliations:** 1 Faculty of Health and Social Sciences, Department of Health Studies, Telemark University College, Porsgrunn, Norway; 2 Department of Occupational and Environmental Medicine, Telemark Hospital, Skien, Norway; 3 Norwegian Institute of Public Health, Division of Environmental Medicine, Oslo, Norway

**Keywords:** tracking, dietary behaviour, eating patterns, principal component analysis, overweight, schoolchildren

## Abstract

**Background:**

The aim of this study was to describe eating patterns in early adolescence and to determine associations between eating patterns and overweight from middle childhood (4^th ^grade, 9 to 10 years old) to early adolescence (7^th ^grade, 12 to 13 years old).

**Methods:**

Children were recruited from primary schools in Telemark County, Norway. Dietary data were obtained by parental report using a food frequency questionnaire. Height and weight were objectively measured, and overweight was defined using international standard cut-off points. Complete data were obtained for 924 4^th ^grade and 691 7^th ^children, and 427 children provided complete data at both time points. Principal component analysis was applied to identify eating patterns. We used multiple logistic regression to calculate adjusted odds ratios (OR) and 95% confidence intervals (CI) for being overweight.

**Results:**

The same four distinct eating patterns were identified at both time points. Correlation coefficients for the factor scores of corresponding eating patterns at baseline and follow up ranged from 0.44 to 0.60. In the follow-up sample, 345 children (80%) were still of normal weight, while 41 (10%) remained overweight. Children with high "dieting" pattern scores and low "varied Norwegian" pattern scores in the 7^th ^grade had an increased risk of being overweight. Children with stable or increased "varied Norwegian" pattern scores had a lower risk of remaining overweight over time than children with decreased scores for this pattern; adjusted OR: 0.4 (95% CI: 0.2, 0.8). This pattern included foods and meals close to current dietary guidelines, including vegetables, fruit and unrefined cereal products. We did not observe an increased risk of overweight in children with high "unhealthy" eating pattern scores, termed "snacking" or "junk/convenient" in either cross-sectional or longitudinal analyses.

**Conclusions:**

Slight to moderate stability of eating patterns was observed. Children adhering to a "varied Norwegian" eating pattern were less likely to remain overweight than children with declining adherence to this pattern. Overweight children should be encouraged to eat regular main meals and retain a diverse diet that includes unrefined plant foods, water and fish, rather than fat- and sugar-reduced foods and drinks.

## Background

The construction of dietary patterns is an alternative to the use of single nutrients or food items in studies of children's food intake. Country-specific dietary patterns have recently been identified for children and adolescents in several European countries [1-9]. The dietary pattern approach may illuminate diet-disease associations that are not revealed when single nutrients or food items are used alone [10,11]. Dietary patterns can also be helpful in evaluating adherence to certain diets over time and risk of disease [11]. The most commonly used method for dietary pattern identification is principal component analysis (PCA), which constructs new linear factors by grouping together correlated variables [12].

In epidemiology, tracking is defined as the stability or maintenance of a given variable over time [13]. Accordingly, dietary tracking may be regarded as maintenance of nutrient intakes, food consumption or dietary habits over a certain time period [4]. Food intake and dietary habits normally change during childhood and adolescence due to individual factors like physiological development, changes in parental influence and social and environmental changes [14]. Dietary habits have mainly been tracked in adults or between adolescence and adulthood, while few studies have tracked diet during childhood [15] and from childhood to adolescence [4,6,14,16,17]

It has been difficult to demonstrate a consistent relationship between BMI in children and dietary factors like fat intake or total energy intake in cross-sectional studies and longitudinal observation studies [18-20]. While most cross-sectional studies have found negative associations between energy-rich foods and overweight, some longitudinal studies have reported a positive association between weight gain over time and consumption of high-fat foods and sugar-sweetened drinks [21,22]. A comprehensive review of studies examining the relationship between dietary intakes, eating behaviours and childhood obesity concluded that more research is needed, particularly in the form of studies exploring the joint effect of multiple dietary behaviours [23]. Two recently published studies have documented a positive relationship between energy-dense diets and overweight in children [24,25]. Further longitudinal studies exploring the association between complex diets and overweight in children are needed.

We have previously reported the association between PCA-identified eating patterns and overweight in a cross-sectional study in 9- to 10-year-old children in Telemark County, Norway [3]. The present study is a follow-up of the same children three years later, in early adolescence (7^th ^grade, 12 to 13 years of age). The aim of the study was to a) describe eating patterns in early adolescence and b) to determine the association between eating patterns and overweight from middle childhood to early adolescence.

## Methods

### Subjects and study design

The present data were obtained from a study of primary school pupils in Telemark County, Norway. Data collection took place in the spring of 2007 and spring of 2010, when the children were in primary school grades 4 (9 to 10 year old) and 7 (12 to 13 years old) respectively. The detailed methods for the 4^th ^grade data collection have been described previously [3]. An identical procedure was used for data collection in the 7^th ^grade. In brief, all primary schools in Telemark County were invited to participate in the study at both time points. Of 110 invited schools 70 agreed to participate in the 4^th ^grade data collection and 53 of 104 participated in the 7^th ^grade data collection. In total, written parental consent to inclusion in the study was received for 1,045 out of 1,477 invited children in the 4^th ^grade and 1,095 out of 1,503 invited children in the 7^th ^grade. This represented about half of the county's 4^th ^and 7^th ^grade pupils at the respective time points.

**Figure 1:**
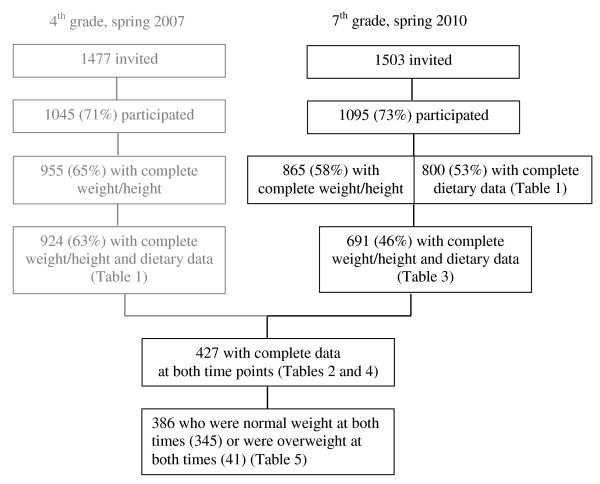
**Overview of participants in the 4^th ^grade (9-10 years) and the 7^th ^grade (12-13 years)**.

Weight and height measurements were obtained for 955 (4^th ^grade) and 865 (7^th ^grade) children, while complete weight/height and dietary data were obtained for 924 and 691 pupils, respectively. In total, 427 children provided complete weight, height and dietary data at both time points (Figure 1).

The research protocol was approved by the Regional Committee for Ethics in Medical Research and the Norwegian Data Inspectorate. Informed written consent was obtained from the parents of all participating children in both 2007 and 2010.

### Dietary information

The children's food and drink intake was reported by their parents using a retrospective food frequency questionnaire (FFQ), which asked about habitual daily consumption of 40 food items, 11 types of drink, 13 types of snacks (between meals) and five main meals (breakfast, lunch, afternoon meal, dinner, supper) during the last six months. Identical FFQs were used at both time points. This questionnaire was based on a short FFQ developed for use among fourth- and eighth-grade children in Norway, but was modified to include more dietary questions. The modified FFQ is appropriate for exploring dietary patterns based on frequencies, but has not been validated for estimating total intakes of energy or nutrients. The alternative frequencies for food and drink items were: "rarely/never", "1-3 times a month", "1-3 times a week", "4-6 times a week", "once a day", "twice a day", and "3 or more times per day". Meal patterns were registered as the daily frequencies of five main meals (breakfast, lunch, afternoon meal, dinner, supper), with 8 response alternatives ranging from "never/rarely" to "daily". The questions about snacking between meals had three answer categories: "never/rarely", "sometimes" and "often/always". As we used meal and snacking events in addition to food consumption frequencies as input variables in the PCA, the components were denoted as 'eating patterns' rather than 'dietary patterns'.

### Other variables

In addition to providing dietary information, the parents answered questions about their own weight, height, educational level and work situation, and family income. They also provided their subjective opinion regarding their child's physical activity level compared with that of other children of the same age, and of time spent on screen-based activities and other sedentary activities outside school (e.g. reading or homework).

Parental educational level was divided into three categories: "primary and lower secondary education" (10 years or less), "upper secondary education" (three to four years of secondary education), and "university or university college".

Family income was divided into three categories: "both parents < Norwegian kroner (NOK) 300,000 (EUR 33,909)", "one parent ≥ NOK 300,000", and "both parents ≥ NOK 300,000".

A variable categorising leisure physical activity by reference to other children was used as an indicator of the children's physical activity level. Parents indicated on a scale from 1-5 whether the child was "less physically active than other children of the same age" or "more physically active compared to other children of same age". The question was taken from a battery of validated questions used in a study of children's activity and inactivity in the Netherlands [26], and translated into Norwegian for use in the present study.

Inactivity was defined as time spent on screen-based activities and other sedentary activities outside school. These activities were combined and divided into two categories: "less than 4 hours per day", and "4 hours or more per day".

### BMI categories

The weight and height of the children were measured by public health nurses at each school at both time points. The children were weighed wearing light clothing (i.e. trousers, T-shirt, socks), using calibrated, electronic scales measuring in 100 g increments. BMI (kg/m^2^) of each child was calculated on the basis of these measurements. Child BMI categories were calculated using International Obesity Task Force (IOTF) cut-off points (underweight, normal weight, overweight, obese), based on growth curves and BMIs of 17, 25 and 30 kg/m^2 ^at age 18 years [27,28]. The respective cut-off points for 9.5-year-old and 12.5-year-old boys and girls were used. Due to small numbers we included underweight children in the normal weight group and obese children in the overweight group.

Changes in BMI categories between the 4^th ^grade and the 7^th ^grade were divided into four categories: "unchanged normal weight", "overweight to normal weight", "normal weight to overweight", and "unchanged overweight". Parent BMI categories were calculated on the basis of self-reported height and weight and the IOTF cut-off points for adults (overweight at BMI ≥ 25 kg/m^2^).

### Statistical analyses

At each of the two time points, we used PCA with varimax rotation to identify eating patterns from the reported dietary responses [29]. Food and drink frequencies were assigned values from 1 for "never/rarely" to 7 for "3 or more times daily", while meal frequencies were assigned values from 1 for "rarely/never" to 8 for "daily" and snacking between meals were assigned 1 for "never/rarely", 2 for "sometimes" and 3 for "often/always". Missing values for a given variable were replaced by rarely/never. Respondents were excluded from the analysis if answers were missing for 16 (23%) or more of the questions about food and drink items or if answers were missing for more than two questions (40%) about meals (n = 31 and n = 68 for the 4^th ^grade and the 7^th ^grade, respectively).

PCA constructs new linear factors by grouping together correlated variables. The coefficients defining the factors are called factors loadings and represent the correlations of each input variable with the factors. The number of components chosen from the factor analysis was based on the scree plot, eigenvalues and the interpretability of the components [29]. The criteria for choosing the components were identical at both time points. Variables with factor loadings > 0.25 or < -0.25 were considered important for interpretability of the components. The components (called eating patterns) were named after the nature of the foods, beverages and meals with the highest factor loadings within each pattern. The four eating patterns previously identified for the children in the 4^th ^grade [3] were: a "snacking" pattern, characterised by snack items and sugar-sweetened drinks, low intake of water, vegetables and brown bread and a low frequency of eating breakfast and dinner; a pattern labelled "junk/convenient", characterised by high-fat and high-sugar processed fast foods; a "varied Norwegian" pattern, characterised by food items typical of a traditional Norwegian diet, close to what is recommended by the health authorities; and, finally, a "dieting" pattern, containing foods and drinks often associated with dieting and weight control.

Individuals were given factor scores for each of the patterns. Factor scores were standardised to a mean of zero. Positive factor scores indicate higher consumption of foods, drinks, snacks and meals in that pattern, while negative factor scores indicate low consumption. The factor scores for each eating pattern were used in the further analysis as continuous variables, or ranked into categorical variables (tertiles).

The factor scores were approximately normally distributed. Therefore, Pearson's correlation coefficients were used to evaluate the agreement between the factor scores for similar and different eating patterns at the two time points. Additionally, Cohen's weighted kappa (κ) [30] was used to compare individual factor scores as categorical variables (tertiles) across time. Cohen's κ for being in the same weight group at both time points was also calculated. In accordance with the scale of Landis and Koch [31], we interpreted the κ-values to represent the following agreement between time points: 0.01 to 0.20: "slight"; 0.21 to 0.4: "fair"; 0.41 to 0.60: "moderate"; 0.61 to 0.80: "substantial"; and 0.81 to 1.00: "almost perfect". BMI was not normally distributed, and Spearman's rho was used for correlation analysis.

Analysis of variance (ANOVA) was used to examine differences in pattern scores between groups. We used multiple logistic regression to calculate adjusted odds ratios (OR) and 95% confidence intervals (CI) for being overweight in the 7^th ^grade and for staying overweight from the 4^th ^to the 7^th ^grade. Categorised pattern scores (low, medium and high, based on tertiles) and a dichotomous variable denoting increase/decrease in pattern scores were used as categorical independent variables in the models, respectively.

We used multiple linear regression and independent samples T-test to examine the changes in eating pattern scores in relation to changes in BMI categories. Changes in eating pattern scores over time were calculated as the difference in pattern scores from the 4^th ^to the 7^th ^grade. The difference within each pattern was examined as the dependent variable, while changes in BMI categories were used as independent variables, with "unchanged normal weight" as the reference category and "overweight to normal weight", "normal weight to overweight", and "unchanged overweight" as independent dichotomous variables.

In order to examine adherence to eating patterns over time, we categorised the changes in eating pattern scores from the 4^th ^to the 7^th ^grade into dichotomous variables denoting either unchanged/increased adherence to the pattern (no change or positive change) or reduced adherence to the pattern (negative change).

The potential confounding variables in the multiple regression models were: maternal and paternal overweight, maternal and paternal education, family income, child physical activity, child sedentary activity, and child gender. We applied a forward conditional selection and included variables significantly associated with overweight in each model. Adjusting for all of the variables had little additional impact on the effect estimates, and led to no changes in the main conclusions in this article.

For all tests, P < 0.05 was considered significant. The questionnaires were scanned by Eyes and Hands (Readsoft Forms, Helsingborg, Sweden), and all the statistical analyses were carried out using SPSS version 15.

## Results

Four distinct eating patterns were identified at both time points (n = 924 and n = 800 for the 4^th ^and the 7^th ^grade, respectively), which were comparable over time (Table 1). The main composition (high-loading items) obtained in the 4^th ^grade was maintained for all four eating patterns in the 7^th ^grade (Table 1). However, some modifications due to changes of items between patterns could be observed, mainly related to items with low factor loadings (between -0.30 and +0.30). A separate PCA performed on the follow-up sample (n = 427) showed only minor differences in eating pattern composition and respective factor loadings compared to the whole sample. The changes observed over time were mainly related to the "varied Norwegian" pattern. Meat for dinner and juice during and between meals, which were part of this pattern in the 4^th ^grade, did not have high loadings for the pattern in the 7^th ^grade, while several main meals and food items were added to the 7^th^-grade pattern: breakfast, lunch, rice, water, jam/honey and margarine/butter on bread (Table 1). Furthermore, low-fat white cheese and yoghurt products, which loaded positively in the "varied Norwegian" pattern in the 4^th ^grade, loaded positively in the "dieting" pattern in the 7^th ^grade. In fact, all yoghurt products loaded positively in the "dieting" pattern in the 7^th ^grade. The main change in the "snacking" pattern was the addition of sugar-sweetened, carbonated soft drinks and "eating between meals" at the 7^th^-grade stage (Table 1). Only small changes were observed for the "junk/convenient" pattern over the time period studied (Table 1).

**Table 1:** Structure of the eating patterns extracted for the 4^th^- (n = 924) and 7^th^-grade (n = 800) children^§^.

	'Junk/convenient'		'Varied Norwegian'		'Snacking'		'Dieting'	
	**4^th ^grade**	**7^th ^grade**	**4^th ^grade**	**7^th ^grade**	**4^th ^grade**	**7^th ^grade**	**4^th ^grade**	**7^th ^grade**

Variance explained by the factor (pattern)	6%	8%	4%	6%	7%	4%	3%	3%

French fries in fast food restaurants	0.60	0.64						
Hamburger or kebab	0.56	0.64						
French fries for dinner	0.49	0.50						
Biscuits, cakes, crackers, etc.	0.51	0.48						
Sausages, hot dog	0.44	0.47						
Processed pizza	0.41	0.46						
Waffles	0.47	0.46						
Sweets	0.32	0.40						
Salty snacks	0.32	0.40						
White bread	0.34	0.40						
Ice cream	0.44	0.39						
Processed meat for dinner	0.26	0.30						
Pancakes	0.45	0.30						
Fruits and berries			0.53	0.59				
Vegetables			0.50	0.58	-0.32			
Fish for dinner			0.40	0.44				
Water				0.44	-0.35			
Brown bread			0.30	0.40	-0.28			
Fruits, berries or vegetables between meals			0.44	0.37		0.28		
Processed fish for dinner			0.32	0.37				
White cheese, full-fat			0.34	0.36				
Cereals without sugar			0.34	0.34				0.28
Brown cheese, full-fat			0.27	0.34				
Water between meals			0.28	0.31				
Low-fat meat on sandwich			0.35	0.31				
Lunch				0.31				
Potatoes, boiled			0.27	0.29				
Breakfast				0.28	-0.31			
Jam, honey as spread				0.28				
Fish spread			0.35	0.27				
Butter or margarine on bread				0.26				
Rice	0.25			0.25				
Non-processed meat for dinner			0.29					
Juice			0.31					
Dinner					-0.28			
Pasta					-0.28			
Sugar-sweetened soft drinks, carbonated (between meals)					0.62	0.59		
Sugar-sweetened soft drinks, non-carbonated (between meals)					0.60	0.58		
Sweets between meals					0.58	0.55		
Juice between meals			0.35		0.43	0.52		0.26
Biscuits, cakes, crackers, etc. between meals	0.34	0.28			0.41	0.50		
Salty snacks between meals		0.25			0.46	0.48		
Ice cream between meals	0.25				0.43	0.45		
Milk between meals					0.47	0.43		
Sugar-sweetened soft drinks, non-carbonated					0.33	0.43	-0.47	-0.33
Yoghurt between meals			0.41		0.43	0.41		0.36
Eating in between meals						0.35		
Sugar-sweetened soft drinks, carbonated	0.34	0.28				0.31	-0.36	-0.29
Artificially sweetened soft drinks, non-carbonated, (between meals)				-0.31	0.30		0.66	0.57
Artificially sweetened soft drinks, carbonated							0.63	0.56
Artificially sweetened soft drinks, non-carbonated		0.25		-0.29			0.65	0.55
Fat- and sugar-reduced yoghurt			0.36				0.29	0.50
Artificially sweetened soft drinks, carbonated (between meals)					0.37		0.54	0.47
White cheese, low-fat			0.33				0.26	0.43
Yoghurt with cereal			0.33			0.25		0.42
Fruit yoghurt			0.45	0.27				0.40
Cereals and breakfast mixtures containing sugar	0.33							
Chocolate spread	0.28							

^§^Food items, snacks and meals with factor loadings > 0.25 or <-0.25 are listed. Patterns are presented by extraction order at the 7^th^-grade stage.

In the 4^th ^grade, the "snacking" pattern explained the largest amount of variance in the data, followed by the patterns "junk/convenient", "varied Norwegian" and "dieting". In the 7^th ^grade however, the "junk/convenient" pattern and the "varied Norwegian" pattern accounted for the largest variations in overall diet. The names or 'labels' that were used to describe the patterns in the 4^th ^grade were highly appropriate to the patterns extracted in the 7^th ^grade, and seemed to describe the composition of the 7^th^-grade patterns even better than the composition of the 4^th ^grade patterns. The "varied Norwegian" pattern was more prominent at the 7^th^-grade stage, as it explained a larger proportion of the variance in intake frequencies and included a larger variety of foods, drinks and main meals.

The Pearson's correlation coefficients for factor scores for the four corresponding eating patterns at the two time points ranged from 0.44 to 0.60 (Table 2). Additionally, the pattern scores for "snacking" and "dieting" in the 4^th ^grade were inversely related to "varied Norwegian" scores in the 7^th ^grade (Table 2). The weighted κ-values for being in the same tertile at the two time points were 0.50, 0.49, 0.37, and 0.44 for the "junk/convenient", "varied Norwegian", "snacking" and "dieting" eating patterns, respectively.

**Table 2:** Correlation coefficients^§ ^for the corresponding eating pattern scores for 4^th^- and 7^th^-grade children (n = 427).

	7th grade 'Snacking'	7th grade 'Junk/convenient'	7th grade 'Varied Norwegian'	7th grade 'Dieting'
	r (95% CI)	r (95% CI)	r (95% CI)	r (95% CI)
4th grade				
'Snacking'	**0.44 (0.36, 0.51)**	0.01 (-0.09, 0.10)	**-0.23 (-0.31, -0.13)**	0.06 (-0.15, 0.04)
'Junk/convenient'	0.06 (-0.04, 0.15)	**0.58 (0.51, 0.64)**	**-0.10 (-0.19, 0.00)**	-0.01 (-0.10, 0.09)
'Varied Norwegian'	0.09 (-0.00, 0.18)	-0.09 (-0.18, 0.01)	**0.60 (0.53, 0.65)**	**0.28 (0.13, 0.32)**
'Dieting'	**-0.16 (-0.25, -0.06)**	0.07 (-0.02, 0.17)	**-0.31 (-0.39, -0.22)**	**0.51 (0.43, 0.57)**

^§ ^Pearsons correlation coefficients (r) and 95% confidence intervals (CI).

At both time points, 50% of the participants were boys and 50% were girls. Weight and height were obtained for 955 (50% boys and 50% girls) of the 1,045 participating children at the 4^th^-grade stage (91%), and for 865 (49% boys and 51% girls) of the 1,095 at the 7^th^-grade stage (79%). For children with complete weight and height measurements at both time points (n = 540), BMI correlated with r = 0.82 (95% CI: 0.79, 0.84). The distribution between normal weight, overweight and obesity was 80%, 16% and 4% in the 4^th ^grade and 81%, 15% and 3% in the 7^th ^grade, respectively. While the incidences of overweight/obesity among boys and girls did not differ in the 4^th ^grade (21% for boys and 20% for girls (p = 0.851)), a significant difference was seen in the 7^th ^grade (21% for boys and 15% for girls (p = 0.001)).

In the follow-up sample (n = 427), 345 (80%) of the children were still of normal weight (48% boys, 52% girls), while 20 (5%) changed from overweight to normal weight (55% boys, 45% girls), 21 (5%) changed from normal weight to overweight (71% boys, 29% girls) and 41 (10%) remained overweight (49% boys, 51% girls). Cohen's Kappa for being in the same weight group at both time points was 0.61 (95% CI: 0.50, 0.72).

At the 7^th^-grade stage (n = 800), the "varied Norwegian" pattern scores were negatively associated with maternal overweight and positively associated with physical activity and with maternal and paternal educational level. The "snacking pattern" scores were positively associated with sedentary behaviour and negatively associated with family income and maternal and paternal educational level. The dieting pattern scores were positively associated with maternal educational level and were higher for girls than boys, while the "junk/convenient" pattern scores were higher for boys than girls (p < 0.05 for all, data not shown).

Cross-sectional analysis of dietary patterns and overweight in the 7^th ^grade showed the highest incidence of overweight in the lower tertile of the "varied Norwegian" pattern (23%) and the upper tertile of the "dieting" pattern (24%) (Table 3). The lowest incidence of overweight was observed in the upper tertile of the "varied Norwegian" pattern (12%) and in the two lower tertiles of the "dieting" pattern (13%). Children ranked in the upper tertile of the "varied Norwegian" pattern were less likely to be overweight than those in the lower tertiles (Table 3), in contrast to what was found in the 4^th ^grade, where high scores for the "varied Norwegian" eating pattern were associated with an increased risk of overweight [3]. Studying the association between single foods and meals and overweight at both time points showed a lower intake of vegetables and less regular breakfast eating among overweight compared to normal weight children at the 7^th ^grade stage.

**Table 3:** Cross-sectional associations^§ ^between tertiles of eating pattern scores and overweight in 7^th ^grade (n = 691).

Eating pattern	n	Overweight and obese children n (%)	OR crude^1 ^(95% CI)	OR adjusted^2 ^(95% CI)
'Junk/convenient'				
Tertile 1	237	42 (18)	1	1
Tertile 2	230	38 (17)	1.0 (0.6, 1.6)	1.0 (0.6, 1.6)
Tertile 3	224	34 (15)	0.8 (0.5, 1.3)	0.8 (0.5, 1.4)
'Varied Norwegian'				
Tertile 1	222	50 (23)	1	1
Tertile 2	232	36 (16)	0.6 (0.4, 1.0)	0.7 (0.4, 1.2)
Tertile 3	237	28 (12)	**0.5 (0.3, 0.8)**	**0.6 (0.4, 0.9)**
'Snacking'				
Tertile 1	222	39 (18)	1	1
Tertile 2	237	37 (16)	0.8 (0.5, 1.3)	0.9 (0.5, 1.5)
Tertile 3	232	38 (16)	0.9 (0.5,1.5)	1.0 (0.6,1.6)
'Dieting'				
Tertile 1	232	30 (13)	1	1
Tertile 2	234	31 (13)	1.0 (0.6, 1.8)	1.1 (0.6, 1.9)
Tertile 3	225	53 (24)	**2.1 (1.3, 3.4)**	**2.2 (1.3, 3.8)**

^§ ^Odds ratios (OR) and 95% confidence intervals (95% CI)

^1 ^Adjusted for the other eating patterns.

^2 ^Adjusted for the other eating patterns and significantly associated background variables.

Independently of this, children ranked in the upper tertile of the "dieting" pattern were more likely to be overweight than those in the lower tertiles in the 7^th ^grade. After adjustment for influential confounders in the model, the associations remained significant (Table 3). No significant associations were observed between either the "junk/convenient pattern" or the "snacking pattern" and overweight in the 7^th ^grade.

Multiple linear regression analysis of the relationship between changes in eating pattern scores and changes in BMI categories over time revealed no significant associations, but weak trends in the data could be observed (Table 4). Compared to children who remained within the "normal weight" category at both time points, children who changed from normal weight to overweight tended to reduce their "junk/convenient" and "snacking" patterns scores and increase their "dieting" pattern scores, while children who changed from overweight to normal weight tended to increase their "snacking" pattern scores. Furthermore, those who were overweight at both time points tended to reduce their scores for the "varied Norwegian" pattern and increase their scores for the "dieting" pattern, compared to the children who stayed normal weight between the 4^th ^and 7^th ^grade (Table 4).

**Table 4:** Changes^§ ^in eating pattern scores relative to change in BMI categories between the 4^th ^and 7^th ^grades.

BMI changes child	Total n = 427 (%)	Change in 'Junk/convenient' Beta (95% CI)	Change in 'Varied Norwegian' Beta (95% CI)	Change in 'Snacking' Beta (95% CI)	Change in 'Dieting' Beta (95% CI)
Unchanged normal weight	345 (81)	Reference	Reference	Reference	Reference
Overweight/obese to normal weight	20 (5)	-0.07 (-0.44, 0.31)	-0.04 (-0.43, 0.35)	0.14 (-0.31, 0.58)	-0.04 (-0.45, 0.37)
Normal weight to overweight/obese	21 (5)	-0.15 (-0.50, 0.19)	-0.07 (-0.43, 0.29)	-0.24 (-0.65, 0.16)	0.13 (-0.25, 0.51)
Unchanged overweight/obesity	41 (10)	-0.11 (-0.39, 0.17)	-0.28 (-0.57, 0.01)	-0.03 (-0.37, 0.30)	0.21 (-0.10, 0.52)

^§ ^Beta coefficients (beta) and 95% confidence intervals (95% CI). All models were adjusted for the other pattern scores and significantly associated background variables.

Finally, we examined the likelihood of remaining in the overweight category over time relative to unchanged/increased adherence to a pattern (no change or positive change in scores between the 4^th ^and 7^th ^grade), or reduced adherence to a pattern (negative change). Children with stable or increased "varied Norwegian" pattern scores showed a significantly lower risk of remaining in the overweight category, compared to children with decreased "varied Norwegian" pattern scores. The association was independent of parental characteristics and child activity/inactivity (Table 5).

**Table 5:** Changes in eating pattern scores and risk^§ ^of remaining overweight between the 4^th ^and 7^th ^grades (n = 386).

Eating pattern change	n	Unchanged overweight n (%)	OR crude^1 ^(95% CI)	OR adjusted^2 ^(95% CI)
'Junk/convenient'				
Decreased scores	201	26 (13)	1	1
Stable or increased scores	185	15 (8)	0.7 (0.3, 1.3)	0.8 (0.4, 1.7)
'Varied Norwegian'				
Decreased scores	172	27 (16)	1	1
Stable or increased scores	214	14 (7)	**0.4 (0.2, 0.8)**	**0.4 (0.2, 0.8)**
'Snacking'				
Decreased scores	169	16 (9)	1	1
Stable or increased scores	217	25 (12)	1.2 (0.6, 2.4)	1.4 (0.7, 2.9)
'Dieting'				
Decreased	174	16 (9)	1	1
Stable or increased scores	212	25 (12)	1.3 (0.6, 2.5)	1.3 (0.6, 2.7)

^§ ^Odds ratio (OR) and 95% confidence intervals (95% CI)

^1 ^Adjusted for the other eating patterns

^2 ^Adjusted for the other eating patterns and significantly associated background variables

## Discussion

The most important finding in the present study was that children adhering to a "varied Norwegian" eating pattern over time had lower risk of remaining overweight than children with declining adherence to this pattern. Cross-sectional analysis of data obtained at the 7^th^-grade stage evidenced a significantly lower risk of being overweight for children with high "varied Norwegian" eating pattern scores, and an increased risk of being overweight for children with high "dieting pattern" scores. Neither cross-sectional nor longitudinal data analysis results indicated an increased risk of overweight for children with high scores for eating patterns characterised by the frequent intake of unhealthy food items.

Tracking was observed for all eating patterns between the two time points. Strong correlation coefficients were observed between factor scores for similar patterns at the two time points, suggesting reasonable stability in individual eating habits over the time period studied. However, significant inverse correlations were observed between two of the 4^th^-grade patterns and "varied Norwegian" in the 7^th ^grade, possibly indicating that children with high 4^th^-grade scores for the "snacking" or "dieting" patterns had lower 7^th^-grade scores for the "varied Norwegian" pattern, and consequently less frequent consumption of the food items included in the dietary guidelines. Likewise, the positive correlation between the "varied Norwegian" pattern in the 4^th ^grade and the "dieting" pattern in the 7^th ^grade could indicate a shift toward more frequent use of fat- and sugar-reduced food and drink items (light products) by many children over the time studied. The weighted κ-values confirmed moderate individual tracking [31] of the "junk/convenient", "varied Norwegian" and "dieting" patterns, and fair tracking of the "snacking" pattern, during the three-year follow-up period. A combination of stability and changes in eating patterns of children and adolescents has also been reported in previous studies [4,6,14,15,32]. The present study supports the proposition that eating habits are tracked from middle childhood into early adolescence, but also emphasise the important changes in children's food habits that occur during this particular period of life.

The correlation between BMI measurements at the two time points was high. In the follow-up sample, the majority of children were still of normal weight (80%), 5% of the children moved from overweight to normal weight, 5% moved from normal weight to overweight and 10% remained overweight. A number of studies have investigated BMI tracking during childhood and adolescence [33,34] and from childhood and adolescence into adulthood [35,36]. The results consistently show a high degree of tracking from childhood to adolescence, a finding supported by the present study.

An important finding in the present study was that children with a high adherence to the 'varied Norwegian' pattern in the 7^th ^grade were less likely to be overweight than children with a low adherence to this pattern, and that children with no change or a positive change in this pattern over time were less likely to remain overweight. It is of particular interest to note that while high scores for the "varied Norwegian" eating pattern were associated with an increased risk of overweight in the 4^th ^grade [3], high scores for the same pattern in the 7^th ^grade were associated with a decreased risk. This change in direction may partly be explained by the changes observed in the composition of the "varied Norwegian" pattern over time, but it is more likely to be explained by individual changes in eating habits by age. Our results showed lower intake of vegetables and less regular breakfast eating among overweight compared to normal weight children at the 7^th ^grade stage. A decreased intake of vegetables and more irregular meal patterns by age have been observed in a nationwide study of Norwegian adolescents [37]. In our study, this trend was only observed among overweight children. The positive association between the "varied Norwegian" pattern and BMI status was evident independent of parental characteristics, gender and physical activity of the child, and corroborates current dietary guidelines which recommend eating a variety of foods including vegetables, fruit, unrefined cereal products and fish and having regular main meals. Regular meals and, especially, regular breakfast intake, have been associated with a reduced risk of overweight in several studies [7,38-42]. A study of food and activity patterns in a longitudinal study of Greek children confirmed that eating frequency, breakfast consumption and adherence to a healthy diet characterised as Mediterranean were negatively associated with overweight [7]. In a study of Norwegian adolescents, in which Telemark was one of two participating counties, eating four (rather than fewer) meals per day was significantly negatively related to being overweight [40].

It has been difficult to demonstrate an increased risk of overweight and obesity for children with a high adherence to unhealthy dietary patterns [5,8,19,23]. However, the British ALSPAC study reported a positive relationship between high-energy diets at ages five and seven and overweight at age nine [24]. In the European DONALD study [25], a small but positive association between consumption of high-energy convenience foods and bodyweight was found among boys. In the present study, we were unable to demonstrate any significant associations with overweight for the eating patterns characterised by frequent intake of unhealthy food items through either cross-sectional or longitudinal data analyses. This may be due to misreporting, including over-reporting of healthy food items and/or under-reporting of unhealthy food items. It is known that food and drink items perceived as 'unhealthy' are under-reported more often than other foods, especially if the children are overweight [43-45]. Another explanation could be that 12- to 13-year-old children eat unhealthy food items when their parents are not aware of it. On the other hand, children that are lean and physically active may tolerate a higher intake of high-energy foods, and are not as likely to under-report these items. Any misreporting is likely to have attenuated the association between eating behaviour and overweight.

Dieting behaviour has been associated with overweight in adolescence [46,47]. In the present study, children with high adherence to a "dieting" pattern were more likely to be overweight than those with low adherence to this pattern in 7^th ^grade as well as in 4^th ^grade. Many so-called "light" products or typical dieting products are intended as substitutes for food items with a high fat or sugar content. Our results did not show any benefit with regard to weight reduction in children having stable or increased scores on the dieting pattern over time. Rather than encouraging weight control mainly through the increased use of fat- and sugar-reduced food products, parents should strive to include more unrefined plant foods, fish, water, and regular meals in their family diet.

The objective measurement of the weight and height of the children at both time points is a major strength of the present study. Self-reported data have been shown to underestimate overweight prevalence in adolescents [48]. Other strengths of the present study include identical data collection at both time points and inclusion of a broad range of background variables, which are likely to capture a significant proportion of the variability in socioeconomic status and health behavior. The longitudinal design and the simultaneous assessment of BMI, diet and physical activity are important in order to evaluate the influence of dietary behavior and physical activity over time. However, our study also has limitations. A problem common to papers using PCA is that the factors are identified based on scree plots, eigenvalues and the interpretability of the components, which involves a good deal of subjectivity. This does not mean that it is not a good method, but the reader must be aware of these limitations. The FFQ did not include portion sizes, and calculating energy and nutrient intakes was not feasible. Consequently, we could not evaluate the validity of the reported intakes based on total energy intake. However, the reproducibility and validity of PCA-derived dietary patterns assessed using FFQs have previously been found to be satisfactory [49]. The FFQ was completed by the parents, and it is likely that the dietary data reflect the parents' "dietary image" rather than the true habitual diet of the children [50]. Parental reporting was chosen to avoid different data collection conditions at the two time points and to reduce under-reporting, which is thought to be common in adolescents [51]. Even if the number of children participating at both time points was reasonably large, there was a loss to follow-up, which was mainly due to incomplete data. The incidence of overweight was lower in the 7^th ^grade than in the 4^th ^grade. The parents did not receive any feedback regarding the child's weight status after the 4^th ^grade study in order to reduce the risk of bias in how parents of overweight children reported the dietary behaviour in the follow-up study. However, we cannot exclude the possibility that the decrease in overweight could be due to underrepresentation of overweight and obese children in the follow-up study, especially among girls. Greater focus on body image and weight control [51] in adolescence may be a reason for lower participation by overweight girls in the 7^th ^grade.

To provide a better understanding of the longitudinal relationship between diet and overweight in adolescents, additional research is needed. We intend to repeat the study when the children reach the age of 15 to 16 years.

## Conclusions

We observed slight to moderate stability of eating patterns between middle childhood and early adolescence, but important changes in individual eating habits were also observed. Children adhering to a "varied Norwegian" eating pattern over time were less likely to remain overweight than children with declining adherence to this pattern. The association was evident independent of parental characteristics, gender and the physical activity levels of the children. Our results indicate that in order to reach normal weight, overweight children should be encouraged to eat regular main meals and retain a diverse diet including unrefined plant foods, water and fish, rather than fat- and sugar-reduced foods and drinks.

## Abbreviations

BMI: Body Mass Index; FFQ: Food Frequency Questionnaire; PCA: Principal Component Analysis; IOTF: International Obesity Task Force; CI: Confidence intervals.

## Competing interests

The authors declare that they have no competing interests.

## Authors' contributions

IMO and MVS were responsible for the study design and data collection. MVS was responsible for the statistical analyses. ALB and IMO assisted in the statistical analyses, and ALB contributed to the interpretation of results. IMO drafted the manuscript. All authors read and approved the final manuscript.
